# The Clinical Significance of Fetal Intra‐Abdominal Umbilical Vein Varix: A Comparative Study

**DOI:** 10.1002/jum.70048

**Published:** 2025-09-18

**Authors:** Keren Zloto, Ariella Wiener, Natalie Bibar, Raanan Meyer, Abeer Massarwa, Michal Fishel Bartal, Alina Weissmann‐Brenner, Eran Kassif, Tal Weissbach

**Affiliations:** ^1^ Department of Obstetrics and Gynecology Sheba Medical Center Tel‐Hashomer Israel; ^2^ School of Medicine, Faculty of Medical and Health Sciences Tel‐Aviv University Tel‐Aviv Israel; ^3^ Department of Obstetrics and Gynecology Hadassah Medical Center, Hebrew University Faculty of Medicine Jerusalem Israel; ^4^ Division of Minimally Invasive Gynecology Cedars Sinai Medical Central Los Angeles CA USA; ^5^ The Bornstein Talpiot Medical Leadreship Program Sheba Medical Center Ramat‐Gan Israel; ^6^ Obstetrics and Gynecology Ultrasound Unit Sheba Medical Center Tel‐Hashomer Israel

**Keywords:** fetal intra‐abdominal umbilical vein varix, neonatal asphyxia, non‐reassuring fetal heart rate, prenatal diagnosis, stillbirth

## Abstract

**Objectives:**

To clarify the clinical significance and optimal management of fetal intra‐abdominal umbilical vein varix (FIUVV).

**Methods:**

A retrospective study comparing composite asphyxia‐related adverse outcomes including stillbirth, cesarean delivery due to non‐reassuring fetal heart rate (CD NRFHR), Apgar <7, Cord pH <7, neonatal intensive care unit admission, mechanical ventilation, seizures, asphyxia, and hypoxic ischemic encephalopathy, as well as rates of small for gestational age (SGA) and congenital anomalies, between FIUVV singletons and the general population born at a single center. A subgroup analysis included FIUVV singletons and controls delivering ≥39 weeks.

**Results:**

Compared to controls (99,715), FIUVV subjects (142) had more congenital anomalies (15.5% versus 0.84%, *P* < .01) and SGA (9.9% versus 5.4%, *P* = .02). There were no stillbirths among FIUVV. There were similar rates of CD NRFHR and asphyxia‐related composite adverse outcomes between the study and control groups (4.2% versus 4.4%, *P* = .9; 7.7% versus 8.7%, *P* = .7) including the subgroup delivering ≥39 weeks (12.5% versus 4.6%, *P* = .06; 12.5% versus 5.7%, *P* = .09).

**Conclusions:**

FIUVV does not appear to increase asphyxia‐related adverse outcomes. Targeted anomaly scan and growth assessment are recommended. There is no evidence to support labor induction before 39 weeks.

**Abbreviations**
AGA: appropriate for gestational age
BMI: body mass index
CD NRFHR: cesarean delivery due to non‐reassuring fetal heart rate
CMA: chromosomal microarray
EFW: estimated fetal weight
FGR: fetal growth restriction
FHRM: fetal heart rate monitor
FIUVV: fetal intra‐abdominal umbilical vein varix
HIE: hypoxic–ischemic encephalopathy
MSAF: meconium‐stained amniotic fluid
NICU: neonatal intensive care unit
PROM: premature rupture of membranes
SGA: small for gestational age

Fetal intra‐abdominal umbilical vein varix (FIUVV) is a fetal venous malformation with a reported incidence of 0.4–1.1/1000.^1^, ^2^ FIUVV is defined as a focal dilatation of the umbilical vein of 9 mm and above, or greater than 2 SD above the mean diameter for gestational age.^1^ The sonographic appearance of a FIUVV is an echolucent cystic dilation at any point along the intra‐abdominal umbilical vein. Color Doppler assists in distinguishing a FIUVV from an intra‐abdominal cyst and, also, demonstrates its turbulent pattern of blood flow, when it occurs.^2^, ^3^ The clinical significance of FIUVV is debated in the literature. Earlier reports have shown FIUVV to be associated with stillbirth, chromosomal abnormalities, fetal hydrops, and other adverse outcomes.^4^, ^5^, ^6^ More recent studies have shown better outcomes, especially in isolated FIUVV.^1^, ^4^, ^7^, ^8^, ^9^, ^10^, ^11^

The current literature is limited, consisting of case series^1^, ^2^, ^4^, ^6^, ^7^, ^8^, ^9^, ^10^, ^11^, ^12^, ^13^, ^14^ and case reports,^5^, ^15^, ^16^, ^17^ the largest of which included 114 cases.^10^ Apart from a study by Koorn et al consisting of 43 cases of isolated FIUVV,^18^ none of the previous studies have compared the outcomes in FIUVV to that of the general fetal population.

This study aimed to compare the clinical characteristics and perinatal outcome in fetuses with FIUVV, isolated and non‐isolated, to those of the general fetal population.

## Methods

**Figure 1:**
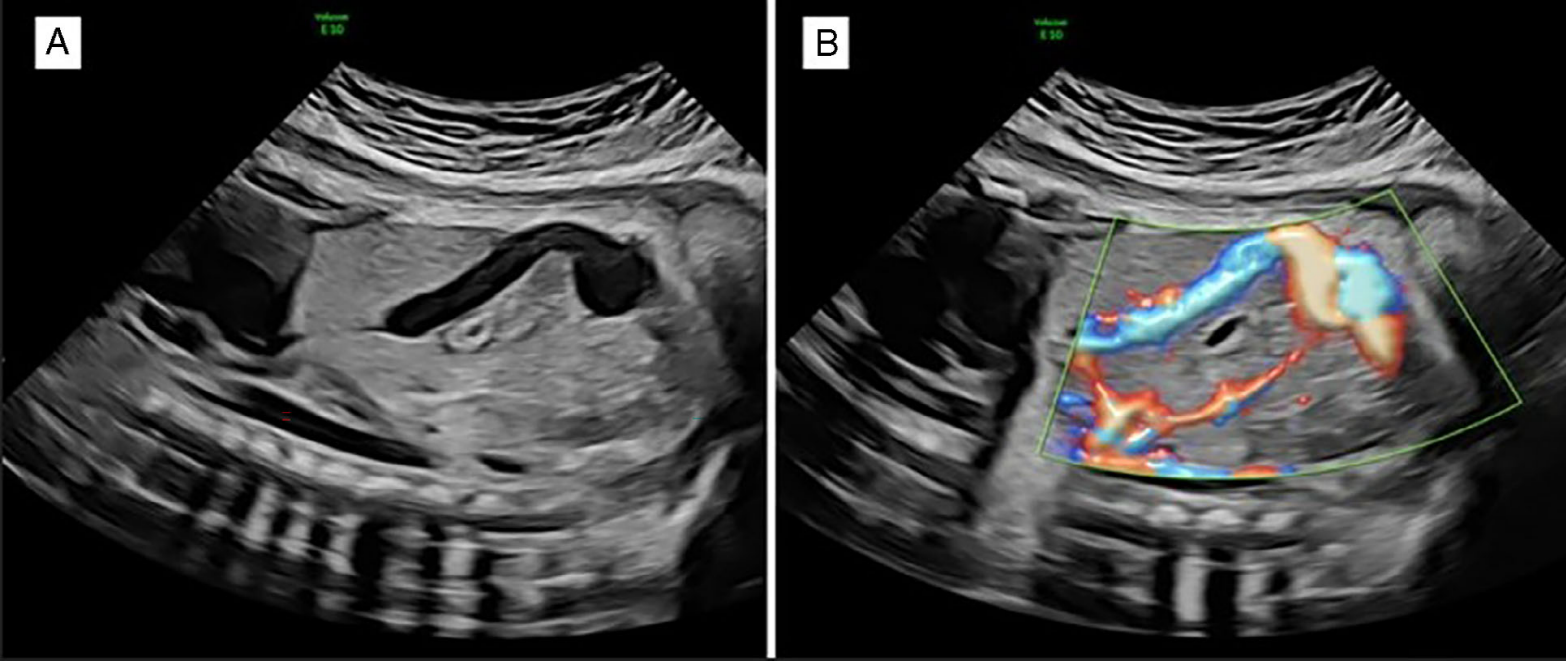
Fetal intra‐abdominal umbilical vein varix on 2D ultrasound (**A**) greyscale (**B**) with power doppler.

**Figure 2:**
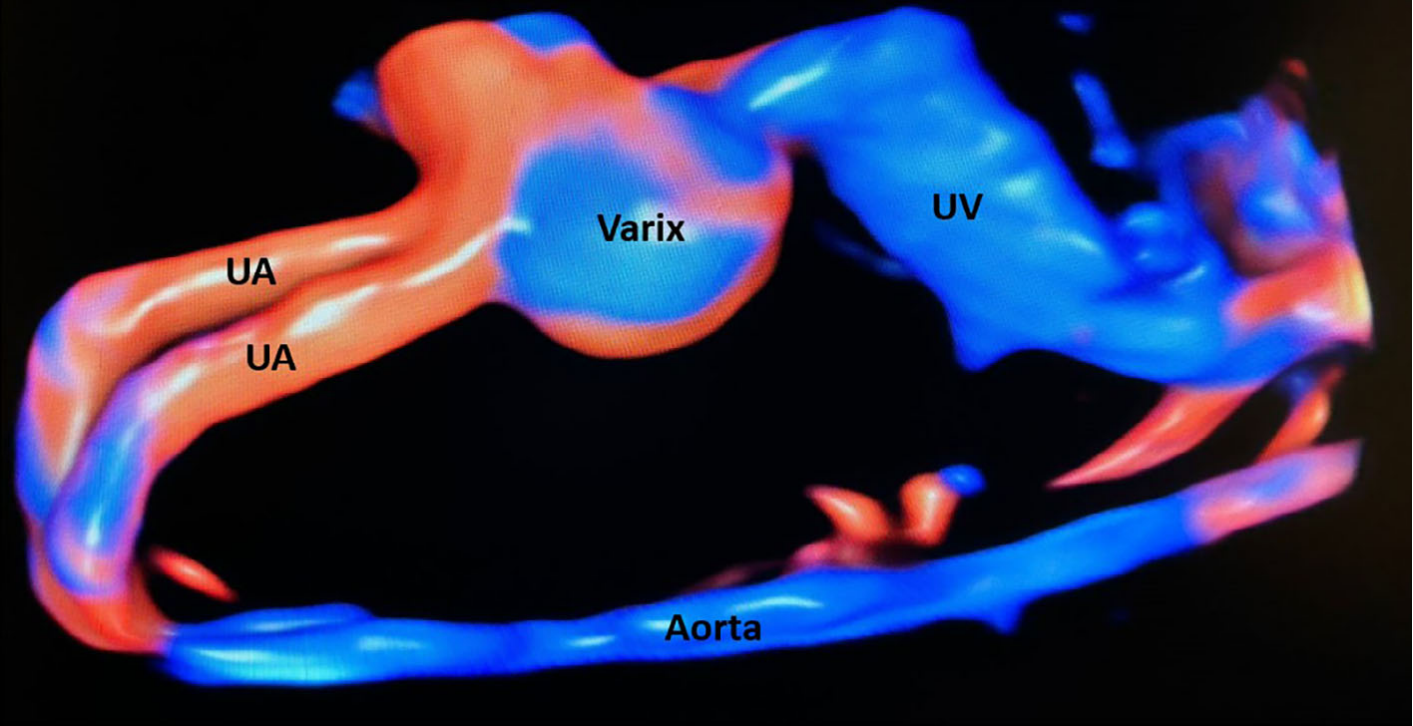
Sagittal view of the fetal abdominal vascular system demonstrating a varix in the umbilical vein on 3D ultrasound. UA, umbilical artery; UV, umbilical vein.

This retrospective study analyzed singleton pregnancies with a diagnosis of FIUVV, all of which delivered at a single tertiary care center between 2012 and 2022. FIUVV was defined as a focal dilation of the intraabdominal portion of the umbilical vein of 9 mm or above, measured on gray scale ultrasound without color Doppler^13^ in the axial plane that demonstrated its widest diameter, placing the calipers on the inner–inner aspect of the vessel walls (Figures 1 and 2). A control group of singleton pregnancies not diagnosed with FIUVV and delivering at our center during the same period was created. Excluded from the study group were cases in which FIUVV was subsequently ruled out by measurement of the umbilical vein below 9 mm on at least 2 occasions after the initial diagnosis was made. Excluded from both groups were high‐order pregnancies and pregnancies that did not deliver at our center.

**Figure 3:**
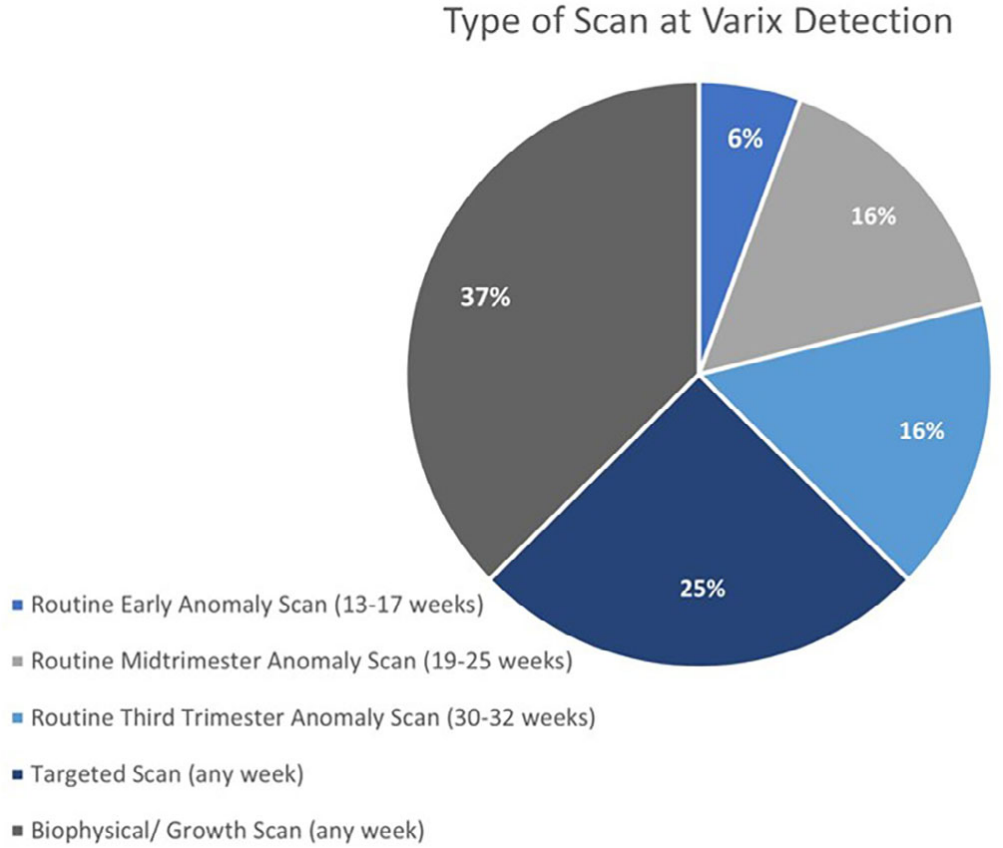
The type of scan at which varix was detected.

FIUVV was diagnosed on either a routine or targeted scan (Figure 3). The Israeli Guideline for Ultrasound in Pregnancy recommends performing a routine early anomaly scan between 13 and 17 weeks of gestation and a mid‐trimester anomaly scan between 19 and 25 weeks of gestation.^19^ It should be noted that the Israeli guideline for mid‐trimester anomaly scan is similar to the ISUOG Practice Guideline for the performance of the routine mid‐trimester fetal ultrasound scan.^20^ A targeted scan is performed when a suspected abnormality arises, such as FIUVV, at any gestational age.

During the study period, the departmental protocol for pregnancies diagnosed with FIUVV included a weekly fetal heart rate monitor (FHRM) and biophysical profile on ultrasound, which assessed fetal movements, tone, breathing movements, and amniotic fluid volume; a fetal growth scan was performed every 2 weeks, and labor was induced at early term (36–38 weeks) or upon detection at term. In case of fetal growth restriction (FGR), Doppler interrogation of the umbilical artery, middle cerebral artery, and ductus venosus was additionally performed.^20^, ^21^ Since increased surveillance was practiced routinely in FIUVV pregnancies, the common finding of turbulent flow in the vessel did not alter the management protocol.

### Perinatal Outcome Measures

A composite asphyxia‐related adverse outcome composed of stillbirth, cesarean delivery due to non‐reassuring fetal heart rate (CD NRFHR), Apgar <7, Cord pH <7, neonatal intensive care unit (NICU) admission, mechanical ventilation, seizures, asphyxia, and hypoxic–ischemic encephalopathy (HIE), was compared between the groups, as well as each outcome, individually.

Other outcomes compared between the groups included the following: gestational age at birth, birthweight, mode of delivery, estimated fetal weight (EFW) <10th centile, small for gestational age (SGA), preterm birth (<37 weeks of gestation), associated anomalies, non‐reassuring fetal heart rate (NRFHR), meconium‐stained amniotic fluid (MSAF), and cesarean delivery (CD). Associated anomalies in both groups included those that were diagnosed prenatally and postnatally, during the postnatal hospital stay.

### Definitions

*Neonatal asphyxia* ^22^ was defined as organ impairment presenting immediately after birth caused by compromised tissue oxygenation resulting from a suspected prenatal hypoxic event, characterized by NRFHR, a low APGAR score, or a low cord pH.

*HIE* ^22^ was defined as neurological impairment appearing immediately after birth resulting from suspected asphyxia with 1 or more of the following presentations: low Apgar score (<7), seizures, reduced tone, decreased reflexes, depressed respiratory effort, or decreased level of consciousness.

Both asphyxia and HIE were diagnosed by the attending neonatologists.

*SGA* was defined as birthweight below 10th centile by Israeli birthweight normal charts.^23^

*EFW* <10th centile according to Hadlock normal growth curves.^24^

### Subgroup Analyses

#### The Significance of Isolated Varix

A subgroup analysis comparing all previously mentioned outcomes in pregnancies carrying non‐anomalous, appropriate for gestational age (AGA) singletons in both groups was conducted to investigate the clinical significance of isolated FIUVV.

#### The Significance of the Maximal Size of Varix

Using the maximal diameter measured in each case, a comparison of perinatal outcomes was performed comparing fetuses with smaller (<12 mm) and larger (≥12 mm) varices to assess the significance of the maximal size of varix.

#### Late Term Delivery

A comparison of perinatal outcomes was conducted between singletons with FIUVV and controls, delivering after 39 weeks, to determine whether FIUVV carries an increased risk for adverse outcomes, beyond the background risk of the general population, at late term.

### Statistical Analysis

The normality of the data was tested using the Shapiro–Wilk or Kolmogorov–Smirnov tests. Continuous variables are presented as medians and interquartile ranges (IQRs), and categorical variables are presented as numbers (%). Comparisons between unrelated variables were conducted using Student's *t* test or the Mann–Whitney *U* test, as appropriate. The chi‐square and Fisher's exact tests were used for comparison between categorical variables.

Significance was determined at *P* < .05. Statistical analyses were conducted using the IBM Statistical Package for the Social Sciences (IBM SPSS v.25; IBM Corporation Inc., Armonk, NY, USA).

The study protocol was approved by the Institutional Ethics Committee at Chaim Sheba Medical Center (approval number 5345‐18‐SMC). Informed consent was waived due to the retrospective design of the study.

## Results

During the study period, 142 singletons diagnosed with FIUVV were included in the study, and 99,715 singletons without FIUVV comprised the control group. We excluded 72 cases: 13 were ruled out on subsequent assessment, 12 were high‐order pregnancies, and 47 delivered at other centers. There were no differences found in the maternal and pregnancy characteristics between patients that delivered at our medical center and those that opted to deliver elsewhere (Table S2).

Most of the cases were detected on a routine scan (6% on early anomaly scan, 16% on mid‐trimester anomaly scan, 16% on third trimester anomaly scan and 37% on a routine biophysical/growth scan). A quarter were detected on a targeted scan for an anomaly or a condition (Figure 3), including suspected CNS (n = 15), genitourinary (n = 6), gastrointestinal (n = 2), and cardiovascular (n = 1) anomalies, amniotic fluid abnormality (n = 2), increased nuchal translucency (n = 2), and vasa previa (n = 1).

Tables 1, 2, 3 compare the background characteristics, perinatal and delivery outcomes between the groups. There were no statistically significant differences between the groups regarding maternal age, body mass index (BMI), nulliparity, gestational diabetes, or hypertensive disorders. Pregnancies with FIUVV had an increased rate of labor induction (62.7% versus 25%, *P* < .01), mostly due to varix (70.7%), while the remaining 29.2% were due to other obstetrical indications, such as NRFHR, premature rupture of membranes (PROM), post term, or oligohydramnios. Patients carrying a fetus with FIUVV delivered at an earlier gestational age (37.9 versus 39.4, *P* < .01) and had a higher rate of preterm birth (15.5% versus 7.2%, *P* < .01) compared to the control group. There were 22 preterm births, most of which were late preterm. Two cases occurred at 33 and 34 weeks of gestation due to preeclampsia and severe FGR, respectively, while the others occurred around 36 weeks. Of these, 5% (1) were spontaneous, and 95% (19) were induced for the following reasons: Varix (n = 11), FGR (n = 4), reduced fetal movements (n = 1), and NRFHR (n = 3). Although the rate of NRFHR was significantly higher in the study group (12% versus 5.4%, *P* = .01), there were similar rates of CD due to NRFHR and composite asphyxia‐related adverse outcomes in both groups (4.2% versus 4.4%, *P* = .9 and 7.7% versus 8.7%, *P* = .7, respectively).

**Table 1:** Comparison of Background Characteristics

	Fetal Varix, n = 142	Control Group, n = 99715	*P* Value	Isolated Fetal Varix,^a^ n = 110	Isolated Control,^a^ n = 93539	*P* Value
Age (years)	32 (29–35.3)	32 (29–36)	.5	31.5 (29–35.3)	32 (29–36)	.9
Nulliparity	41 (28.9%)	35,851 (36%)	.08	33 (30%)	32,701 (35%)	.3
BMI (kg/m^2^)	22 (20.2–26.7)	22.4 (20.2–25.4)	.9	22 (20.2–26)	22.5 (20.3–25.4)	.9
IVF	15 (10.6%)	16,795 (16.8%)	<.05	12 (10.9%)	15,556 (16.6%)	.1
Gestational diabetes	16 (11.3%)	10,088 (10.1%)	.6	10 (9.1%)	9543 (10.2%)	.7
Hypertensive disorder	6 (4.2%)	3482 (3.5%)	.6	3 (2.7%)	3009 (3.2%)	.8
VBAC^b^	6 (4.2%)	4754 (4.8%)	.8	5 (4.5%)	4521 (4.8%)	.9

Data are presented as median (interquartile range) or n (%).

BMI, body mass index; IVF, in vitro fertilization; VBAC, vaginal birth after cesarean section.

^a^ Without anomalies or fetal growth restriction.

^b^ Among patients after 1 cesarean section.

**Table 2:** Comparison of Perinatal and Asphyxia‐Related Adverse Outcomes in Varix and Isolated Varix

	Fetal Varix, n = 142	Control, n = 99,715	*P* Value	Isolated Fetal Varix,^a^ n = 110	Isolated Control,^a^ n = 93,539	*P* Value
GA at delivery (weeks)	37.9 (37.1–38.8)	39.4 (38.4–40.3)	<.01	38.1 (37.1–39)	39.4 (38.4–40.3)	<.01
Birthweight (g)	3193 (2753–3516)	3230 (2925–3530)	.02	3235 (2861–3570)	3260 (2985–3550)	.2
Small for gestational age	14 (9.9%)	5396 (5.4%)	.02	N/A	N/A	N/A
EFW <10th centile	18 (12.7%)	2884 (2.9%)	<.01	N/A	N/A	N/A
Additional anomalies	22 (15.5%)	842 (0.8%)	<.01	N/A	N/A	N/A
Preterm labor	22 (15.5%)	7181 (7.2%)	<.01	12 (11%)	6235 (6.7%)	.08
Asphyxia‐related composite adverse outcome^b^	11 (7.7%)	8633 (8.7%)	.7	7 (6.4%)	7190 (7.7%)	.6
Stillbirth	0 (0%)	1043 (1%)	.2	0 (0%)	859 (0.9%)	.3
NRFHR	17 (12%)	5441 (5.4%)	.01	14 (12.7%)	4623 (4.9%)	<.01
Cesarean section due to NRFHR	6 (4.2%)	4354 (4.4%)	.9	5 (4.5%)	3630 (3.9%)	.6
Meconium‐stained amniotic fluid	4 (2.8%)	11288 (11.3%)	<.01	3 (2.7%)	10642 (11.4%)	<.01
Apgar_5 min_ <7	1 (0.7%)	347 (0.3%)	.4	1 (0.9%)	292 (0.3%)	.3
Cord pH <7	0 (0%)	226 (0.2%)	.6	0 (0%)	200 (0.2%)	.8
Neonatal asphyxia	0 (0%)	79 (0.1%)	.7	0 (0%)	68 (0.1%)	.8
Hypoxic ischemic encephalopathy	0 (0%)	104 (0.1%)	1	0 (0%)	90 (0.1%)	.9
Mechanical ventilation	1 (0.7%)	910 (0.9%)	1	1 (0.9%)	760 (0.8%)	.6
Respiratory distress syndrome	1 (0.7%)	112 (0.1%)	.1	1 (0.9%)	104 (0.1%)	.1
Seizures	1 (0.7%)	114 (0.1%)	.2	0 (0%)	92 (0.1%)	.9
NICU admission	6 (4.2%)	3442 (3.4%)	.6	2 (1.8%)	2721 (2.9%)	.8
Neonatal death during admission	0 (0%)	46 (0.04%)	1	0 (0%)	32 (0.03%)	1

Data are presented as median (interquartile range) or n (%).

GA, gestational age; FGR, fetal growth restriction; NRFHR, non‐reassuring fetal heart rate; NIUC, neonatal intensive care unit.

^a^ Without anomalies or fetal growth restriction.

^b^ Including fetal death, operative vaginal/CS delivery due to NRFHR, Apgar_5_ score <7, umbilical cord pH <7, neonatal asphyxia, hypoxic ischemic encephalopathy, mechanical ventilation, NICU admission, and neonatal death before discharge.

**Table 3:** Delivery Outcomes for Varix and Isolated Varix

	Fetal Varix, n = 142	Control Group, n = 99,715	*P* Value	Isolated Fetal Varix,^a^ n = 110	Isolated Control,^a^ n = 93,539	*P* Value
Mode of delivery						
NVD	106 (74.6%)	68.6% (68420)	.1	75.45 (83%)	64743 (69.2%)	.2
Operative vaginal	4 (2.8%)	6396 (6.4%)		3 (2.7%)	5882 (6.3%)	
CS^b^	32 (22.5%)	24899 (25%)		23 (20.9%)	22914 (24.5%)	
Labor induction	89 (62.7%)	24928 (25%)	**<.01**	65 (59.1%)	22696 (24.3%)	**<.01**

Data are presented as median (interquartile range) or n (%). Bold font indicates statistically significant differences.

NVD, normal vaginal delivery; CS, cesarean section.

^a^ Isolated = excluding anomalies and fetal growth restriction.

^b^ CS indications: Malpresentation, previous cesarean section, maternal request, severe FGR, macrosomia, suspected placenta accreta, non‐reassuring fetal heart rate, and suspected cephalopelvic disproportion.

Fetuses with FIUVV had a higher rate of SGA (9.9% versus 5.4%, *P* = .02), EFW <10th centile (12.7% versus 2.9%, *P* < .01) and congenital anomalies (15.5% versus 0.8%, *P* < .01) compared to the control group. In the FIUVV group, all cases with an EFW <10th centile fulfilled the Delphi consensus definition of FGR. Notably, fetuses with an EFW >10th centile were also assessed for FGR according to the consensus definition but did not meet criteria.^25^ In the control group, data on Doppler interrogation were missing; therefore, the rate of FGR in this group is unknown. There were no cases of stillbirth in the FIUVV group (0% versus 1%, *P* = .2).

Table 4 elaborates the type of structural and genetic anomalies found in the FIUVV group. Overall, 22 cases (15.5%) had additional anomalies, most commonly in the central nervous system and genitourinary system. In 16 cases, FIUVV was diagnosed following the detection of an anomaly on a previous scan. In 5 cases, the anomalies were diagnosed on the same scan as the FIUVV. In 1 case, the anomaly was diagnosed after the FIUVV, on a routine scan. Genetic investigation was performed in 37.3% (53/142) of the FIUVV cases; 47 performed chromosomal microarray (CMA), 6 performed karyotyping, and 3 performed exome sequencing in addition to CMA. The type of genetic testing was determined by what was available at the time. CMA has been commercially available since 2013 and exome sequencing since 2020. All of the structural and genetic abnormalities presented in Table 4 were detected prenatally. No additional abnormalities were detected postnatally up to the point of hospital discharge.

**Table 4:** Classification of Associated Anomalies and Genetic Abnormalities Among 22 Non‐isolated Cases

Anatomical System	List of Anomalies	
Central Nervous	Dysgenetic corpus callosum (2), unilateral ventriculomegaly (3), microcephaly (2)	
Cardiovascular	Aberrant right subclavian artery (1), ventricular septal defect (1), atrio‐ventricular septal defect (1), single umbilical artery (1)	
Gastrointestinal	Porto‐systemic shunts (2), annular pancreas (1)	
Genito‐urinary	Ectopic kidney (2), bilateral hydronephrosis (2), unilateral undescended testes (1)	
Craniofacial	Micro/retrognathia (1), congenital torticollis (2)	
Genetic abnormalities		
Abnormal karyotype	0/6 (0%)	N/A
Abnormal chromosomal microarray	3/47 (6.4%)	MID1‐related Opitz G/BBB syndrome (1) Trisomy 21 (1), 2q11.1 duplication (1)
Abnormal whole exome sequencing	0/3 (0%)	N/A

Genetic aberrations were found in 3 patients, all of whom were anomalous, rendering a 13.6% (3/22) rate of genetic abnormality among non‐isolated FIUVV and 0% among isolated FIUVV.

### Isolated FIUVV

Tables 1, 2, 3 present the comparison of the background characteristics, perinatal and delivery outcomes between isolated FIUVV (n = 110) and its corresponding control group (n = 93,539). The isolated FIUVV delivered significantly earlier than the control group (38.1 versus 39.4 weeks, *P* < .01); however, the difference in the rate of preterm birth did not reach statistical significance (11% versus 6.7%, *P* = .08). Among the 12 cases of preterm labor in the study group, 1 was spontaneous while 11 were induced for: Varix (n = 8), NRFHR (n = 2) and reduced fetal movements (n = 1). The study group had a higher rate of labor induction (59.1% versus 24.3%, *P* < .01) and a similar distribution of mode of delivery as the control group.

Although there was a higher rate of NRFHR (12.7% versus 4.9%, *P* < .01) in the study group compared to the control group, there were similar rates of CD due to NRFHR and asphyxia‐related composite adverse outcome between the groups (4.5% versus 3.9%, *P* = .6 and 6.4% versus 7.7%, *P* = .6, respectively). There was a lower rate of MSAF (2.7% versus 11.4%, *P* < .01) compared to controls.

### The Significance of the Maximal Size of the Varix

Table 5 presents the comparison of perinatal outcomes between 35.2% (50) cases of FIUVV measuring <12 mm and 64.8% (92) cases measuring ≥12 mm. The only difference detected was a higher rate of associated anomalies in the ≥12 mm group (20.7% versus 6%, *P* = .02). There were no statistically significant differences detected in the remaining perinatal outcomes, including rates of preterm birth (10% versus 18.5%, *P* = .2), SGA (16% versus 6.5%, *P* = .8), NRFHR (10% versus 13%, *P* = .6), CD due to NRFHR (6% versus 3.3%, *P* = .7) and asphyxia‐related composite adverse outcome (6% versus 8.7%, *P* = .7).

**Table 5:** Comparison of Characteristics of Fetuses with Varix Measuring Below and Above 12 mm

	Varix Maximal Diameter <12 mm, n = 50	Varix Maximal Diameter ≥12 mm, n = 92	*P* Value
Gestational age at delivery (weeks)	38 (37.1–39.2)	37.7 (37.1–38.6)	.9
Week at diagnosis of maximal varix size	35.6 (32–37)	34.6 (33–36.6)	.2
Associated anomalies	3 (6%)	19 (20.7%)	.**02**
Preterm birth (<37 weeks)	5 (10%)	17 (18.5%)	.2
Labor induction	33 (66%)	57 (62%)	.7
Cesarean delivery	9 (18%)	22 (23.9%)	.4
NRFHR	5 (10%)	12 (13%)	.6
Cesarean delivery due to NRFHR	3 (6%)	3 (3.3%)	.7
Stillbirth	0 (0%)	0 (0%)	N/A
SGA	8 (16%)	6 (6.5%)	.08
Meconium‐stained amniotic fluid	2 (4%)	2 (2.2%)	.6
Apgar_5 min_ <7	1 (2%)	0 (0%)	.5
Cord Ph <7	0 (0%)	0 (0%)	N/A
Asphyxia	0 (0%)	0 (0%)	N/A
NICU admission	1 (2%)	5 (5.4%)	.4
RDS	0 (0%)	1 (1.1%)	1
Mechanical ventilation	0 (0%)	1 (1.1%)	.5
Seizures	1 (2%)	0 (0%)	.4
Neonatal death before discharge	0 (0%)	0 (0%)	N/A
Asphyxia‐related composite adverse outcome^a^	3 (6%)	8 (8.7%)	.7

Data are presented as median (interquartile range) or n (%). Bold font indicate statistically significant differences.

RDS, respiratory distress syndrome; SGA, small for gestational age; NIUC, neonatal intensive care unit; NRFHR, non‐reassuring fetal heart rate.

^a^ Including fetal death, operative vaginal/CS delivery due to NRFHR, Apgar_5_ score <7, umbilical cord pH <7, neonatal asphyxia, hypoxic ischemic encephalopathy, mechanical ventilation, NICU admission, and neonatal death before discharge.

### Late Term Delivery

Table 6 presents the comparison of perinatal outcomes between 32 FIUVV singletons and 62,397 controls delivering ≥39 weeks. Of the 32 FIUVV cases delivered at or beyond 39 weeks, 22 declined early term labor induction, 6 were only detected at this stage, and 4 had a failed early term induction and were, therefore, postponed to a later date. There were no statistically significant differences detected between the groups in the rates of CD, CD due to NRFHR, and of asphyxia‐related composite adverse outcome (12.5% versus 15.1%, *P* = .7, 12.5% versus 4.6%, *P* = .06 and 12.5% versus 5.7%, *P* = .09).

**Table 6:** Perinatal Outcome Comparison of Varix and Controls Born ≥39 Weeks of Gestation

	Varix, n = 32	Control, n = 62,397	*P* value
Age (years)	33 (30.25–36.75)	32 (28–35)	.**04**
Gestational age at delivery (weeks)	39.71 (39.32–40.1)	40.14 (39.57–40.71)	.**03**
Stillbirth	0 (0%)	37 (0.05%)	.9
Non‐isolated	3 (9.4%)	2792 (4.5%)	.18
Associated anomalies	2 (6.25%)	472 (0.75%)	.**02**
SGA	1 (3.1%)	2343 (3.75%)	.85
Cesarean delivery	4 (12.5%)	9443 (15.1%)	.7
Cesarean section due to NRFHR	4 (12.5%)	2848 (4.6%)	.6
NRFHR	8 (25%)	3537 (5.7%)	**<.01**
Meconium‐stained amniotic fluid	1 (3.1%)	9827 (15.7%)	.05
Apgar_5 min_ <7	0 (0%)	115 (0.2%)	.8
Cord Ph <7	0 (0%)	138 (0.2%)	.8
Asphyxia	0 (0%)	41 (0.06%)	.9
NICU admission	1 (3.1%)	640 (1%)	.2
RDS	0 (0%)	14 (0.02%)	1
Mechanical ventilation	0 (0%)	171 (0.3%)	1
Seizures	0 (0%)	47 (0.07%)	1
Neonatal death before discharge	0 (0%)	7 (0.01%)	1
Asphyxia‐related composite adverse outcome^a^	4 (12.5%)	3548 (5.7%)	.09

Data are presented as n (%). Bold font indicate statistically significant differences.

SGA, small for gestational age; RDS, respiratory distress syndrome; NIUC, neonatal intensive care unit; NRFHR, non‐reassuring fetal heart rate.

^a^ Including stillbirth, operative vaginal/CS delivery due to NRFHR, Apgar_5_ score <7, umbilical cord pH <7, neonatal asphyxia, hypoxic ischemic encephalopathy, mechanical ventilation, NICU admission, and neonatal death before discharge.

### FIUVV without Congenital Malformations

Table 1 presents a comparison of perinatal and asphyxia‐related adverse outcomes in FIUVV cases without congenital malformations and controls. The FIUVV group displayed higher rates of PTB, SGA, and NRFHR than the control group (13.3% versus 7.2%, *P* < .01, 11.7% versus 5.4%, *P* < .01 and 11.7% versus 5.4%, *P* < .01, respectively) with similar rates of asphyxia‐related composite adverse outcomes (5.8% versus 8.6%, *P* = .28).

## Discussion

To the best of our knowledge, this is the largest published cohort of FIUVV that compares outcomes in FIUVV with the general fetal population. The main findings of the current study are that FIUVV is associated with SGA and fetal anomalies, but otherwise presents similar mortality and asphyxia‐related adverse outcomes to the general singleton population, indicating an overall good prognosis. Secondly, FIUVV measuring ≥12 mm presented a higher rate of anomalies compared to FIUVV <12 mm, but with a similar asphyxia‐related adverse outcome. Thirdly, there were similar perinatal and asphyxia‐related adverse outcomes in fetuses with and without FIUVV delivering at ≥39 weeks, with no stillbirths among the FIUVV group.

The reported rate of stillbirth in FIUVV has decreased over the years. Earlier reports suggested a stillbirth rate of up to 44%,^2^, ^11^, ^14^, ^26^ perhaps reflecting a publication bias. The pathogenesis of stillbirth is not clear; some suggest umbilical vein thrombosis as a possible explanation.^26^, ^27^ However, more recent, larger studies reported a better prognosis, especially in isolated FIUVV.^1^, ^6^, ^8^, ^9^, ^13^, ^14^, ^15^ The largest case series so far, conducted by Lee et al, comprising 114 FIUVV cases, of which 102 were isolated, reported no stillbirths among the isolated cases and 1 stillbirth among the 19 non‐isolated cases, which occurred at 40 weeks of gestation.^10^ Another relatively large case series by Byers et al reporting on 52 cases of FIUVV presented a single case of stillbirth at 35 weeks, which occurred in an anomalous fetus diagnosed with Down's syndrome.^4^ One anomalous fetus experienced stillbirth in a series of 20 FIUVV cases published by Novoa et al.^6^ In these instances, stillbirth may have been attributed to the co‐existing risk factors of aneuploidy, anomalies, and advanced pregnancy,^28^, ^29^ and not necessarily to the varix itself. Likewise, no stillbirths occurred in a cohort of 43 isolated FIUVV singletons, and only 2 cases of IUFD were attributed to FIUVV in a pooled group of 513 FIUVV cases in a meta‐analysis published by Koorn et al.^18^ In agreement with contemporary literature, there were no stillbirths in our cohort of 142 cases, neither in the isolated nor in the non‐isolated cases, even among those delivering during late term. Contrary to the benign nature of FIUVV, a persistent vitelline vein aneurysm is an extremely rare venous aneurysm with high mortality, which may be erroneously diagnosed as FIUVV.^30^ Due to the different prognosis and management of these conditions, it is crucial to differentiate between these conditions.

Another interesting observation of the current study was the increased rate of NRFHR compared to controls, for both isolated and non‐isolated FIUVV. The underlying pathogenesis of this phenomenon is unknown, and this may reflect a bias due to clinicians' awareness of the FIUVV diagnosis; however, both patients and clinicians can be reassured that these events were clinically insignificant and did not lead to higher rates of emergency CD, stillbirth, or other asphyxia‐related composite adverse outcomes. Additionally, there was a lower rate of MSAF in the study group, providing additional reassurance of fetal well‐being in FIUVV; however, a lower rate of meconium may also be related to the earlier gestational age at the time of delivery in this group.^30^ Furthermore, it appeared in our cohort that the size of the FIUVV did not affect perinatal and asphyxia‐related outcomes. A similarly favorable perinatal outcome has been evidenced in previous reports.^4^, ^10^, ^13^, ^31^

The current study substantiates the suggested association of FIUVV and congenital anomalies. However, while previous studies were merely observational, reporting a rate of up to 35%,^1^, ^3^, ^10^, ^26^, ^31^ our study validated this association by proving statistical significance (15.5% versus 0.84%, *P* < .01). This rate increased in varices measuring ≥12 mm compared to those measuring <12 mm (20.7% versus 6%, *P* = .02). In the face of this consistent observation and despite a possible detection bias, it would be prudent to refer patients for a thorough anomaly scan once FIUVV is detected. The need for fetal echocardiography in addition to a comprehensive scan depends on the experience of the expert sonographer in cardiac assessment, as previous reports suggest a higher rate of cardiac anomalies in FIUVV, some of which are lethal.^31^

This is the first study to report CMA results in FIUVV. There was a 6.4% (3/47) rate of copy number abnormality (Trisomy 21, MID1‐related Opitz G/BBB syndrome and a 2q11.1 duplication), all of which were anomalous, rendering a 13.6% (3/22) rate of CMA abnormality among non‐isolated FIUVV. This is higher than the reported 6% rate of CMA abnormality in the general anomalous fetal population.^32^, ^33^ The literature is inconsistent regarding karyotype abnormality in FIUVV. For example, Lee et al reported no karyotype aberrations among a cohort of 114 patients,^10^ while Byers et al found a 5.8% rate of Trisomy 21 in a cohort of 52 cases.^4^ Other small case series and case reports noted a possible association of karyotype abnormality in FIUVV.^2^, ^3^, ^6^, ^11^, ^12^, ^17^, ^26^ Considering our data as well as these reports, it would be prudent to offer CMA especially in non‐isolated FIUVV cases.

Additionally, we found a relatively high rate of FGR of 12.7% compared to previous reports by Byers et al and Lee et al which reported lower rates of growth restriction (1.9 and 3.5%).^4^, ^10^ In contrast, the studies published by Mankuta et al and Koorn et al found similar FGR rates as our cohort (10.7 and 16.4%, respectively).^8^, ^18^ In light of these observations, it would be cautious to recommend fetal weight surveillance in the 3rd trimester in FIUVV.

Our study provides additional evidence of the excellent perinatal outcome in FIUVV, with no apparent supplemental risk of asphyxia, even among pregnancies delivering at ≥39 weeks. These observations are valuable to the growing body of evidence of the benign nature of isolated FIUVV,^1^, ^10^ which may be considered a variant of the norm, perhaps obviating the need for early labor induction, thereby avoiding unnecessary medical intervention and iatrogenic prematurity and their associated risks.

The current study is not free of limitations. Its main limitation is the retrospective design and non‐uniform management in both groups. Additionally, the size of the cohort does not provide sufficient statistical power to detect small increments in the rate of some of the outcomes assessed, such as stillbirth, but it is powered to detect an increase of 3% from the 1% stillbirth rate found in our fetal population. A future meta‐analysis pooling large published series may be able to determine smaller increments in the rate of stillbirth, as well as other outcomes. Furthermore, only a small group of FIUVV was available to assess the risk of late‐term pregnancy in FIUVV, limiting its statistical power to confirm possible trends such as higher rates of CD due to NRFHR and asphyxia‐related composite adverse outcomes in FIUVV. This is due to the common practice of early term labor induction, leaving few patients carrying a fetus with FIUVV in the late term to compare to late‐term controls. Despite the small numbers, we find value in this comparison since this finding is novel and may indicate that FIUVV does not carry an increased risk of perinatal morbidity and mortality. Another possible limitation that must be considered is detection bias since some of the FIUVVs were diagnosed following the detection of an anomaly on a previous scan. However, most of the cases were isolated and were detected despite being an isolated finding. When comparing for growth restriction, we were constrained to use SGA and EFW <10th centile as proxies for FGR, an acceptable compromise,^34^ since a large proportion of the control group did not have a fetal weight assessment close to birth or accessible data to Doppler results. Another potential source of selection bias stems from the exclusion of cases that did not deliver at our center. Approximately, 25% of the identified FIUVV cases chose at their discretion to deliver elsewhere. Their exclusion from the analysis could have potentially created a selection bias. However, comparing these groups, no differences in the maternal and pregnancy characteristics were found, decreasing this potential selection bias. Despite these limitations, the study has several strengths worth mentioning, including its precedence as the first study to evaluate perinatal outcomes in FIUVV delivering at late term and the largest study to compare outcomes in FIUVV with the general singleton population, providing novel clinical insights.

## Conclusion

FIUVV carries a good prognosis, especially when isolated. Due to higher rates of growth restriction and congenital anomalies compared to the general fetal population, a targeted anomaly scan and growth assessment are recommended. Genetic investigation should be offered in non‐isolated cases. There is no evidence to support labor induction before 39 weeks in the absence of an obstetrical indication.

## Supporting information


**Table S1.** Comparison of perinatal and asphyxia‐related adverse outcomes between non‐anomalous fetuses with varix and controls, including small for gestational age.
**Table S2.** Comparison of maternal and pregnancy characteristics in FIUVV cases according to delivery location.

## Data Availability

The data that support the findings of this study are available on request from the corresponding author. The data are not publicly available due to privacy or ethical restrictions.
