# Supplementary material for: The Clinical Significance of Fetal Intra‐Abdominal Umbilical Vein Varix: A Comparative Study
Source: J Ultrasound Med. 2025 Sep 18;45(1):181–92. doi: 10.1002/jum.70048 (PMC12669433; doi:10.1002/jum.70048)
Supplement: Supplementary file 1 — Table S1. Comparison of perinatal and asphyxia‐related adverse outcomes between non‐anomalous fetuses with varix and controls, including small for gestational age. Table S2. Comparison of maternal and pregnancy characteristics in FIUVV cases according to delivery location. [file JUM-45-181-s001.docx]

Table 1 s: Comparison of Perinatal and Asphyxia-Related Adverse Outcomes Between Non-Anomalous Fetuses with Varix and Controls, including small for gestational age

|  | Fetal Varix Without Malformations  N= 120 | Controls Without Malformations N=98,873 | P-value |
| --- | --- | --- | --- |
| Gestational age at delivery (weeks) | 37.9(37.1-39) | 39.4(38.4-40.3) | **<0.01** |
| Preterm Birth (<37 weeks) | 16 (13.3%) | 77,114 (7.2%) | **<0.01** |
| Cesarean Delivery | 26 (21.7 %) | 24,628 (24.9%) | 0.41 |
| NRFHR | 14 (11.7 %) | 5,372 (5.4 %) | **<0.01** |
| Cesarean Delivery due to NRFHR | 5 (4.2 %) | 4,298 (4.35%) | 0.92 |
| Stillbirth | 0 (0%) | 1,043 (1 %) | 0.25 |
| SGA | 13 (10.8%) | 5,334 (5.4 %) | **<0.01** |
| Meconium-Stained Amniotic Fluid | 4 (3.3%) | 11,201 (11.3%) | **<0.01** |
| Asphyxia | 0 (0%) | 78 (0.01%)) | 0.75 |
| NICU Admission | 2 (1.7%) | 3,329 (3.4 %) | 0.3 |
| Mechanical Ventilation | 1 (0.8%) | 880 (0.9%) | 0.94 |
| Asphyxia-Related Composite Adverse Outcome* | 7 (5.8%) | 8,482 (8.6%) | 0.28 |

Data are presented as Median (Interquartile Range) or N (%).

GA- Gestational Age; NRFHR- Non-Reassuring Fetal Heart Rate; SGA- Small for Gestational Age; NIUC-neonatal intensive care unit.

^*^ including fetal death, operative vaginal/CS delivery due to NRFHR, Apgar5 score < 7, umbilical cord pH <7, neonatal asphyxia, hypoxic ischemic encephalopathy, mechanical ventilation, NICU admission and neonatal death before discharge.

Table 2 s: Comparison of Maternal and Pregnancy Characteristics in FIUVV Cases According to Delivery Location

|  | Delivered at Sheba Medical Center  N= 142 | Delivered Elsewhere  N=47 | P-value |
| --- | --- | --- | --- |
| Age (Years) | 32  (29-35.3) | 31(27.5-34) | 0.06 |
| Nulliparity | 41 (28.9%) | 7 (14.9%) | 0.14 |
| In vitro fertilization | 15 (10.6%) | 0 (0%) | 0.06 |
| Gestational Diabetes | 6 (4.23%) | 0 (0%) | 0.62 |
| Hypertensive Disorder | 6 (4.23%) | 0 (0%) | 1 |

Data are presented as Median (Interquartile Range) or N (%).
